# Clinical and Radiographic Mid-Term Outcomes After Total Shoulder Replacement: A Retrospective Study Protocol Including 400 Anatomical and Reverse Prosthetic Implants

**Published:** 2016-05-16

**Authors:** Giovanni Merolla, Antonio Tartarone, Giuseppe Porcellini

**Affiliations:** 1 Unit of Shoulder and Elbow Surgery, D. Cervesi Hospital, Cattolica, AUSL della Romagna Ambito Territoriale di Rimini - Italy; 2 Biomechanics Laboratory “Marco Simoncelli” D. Cervesi Hospital, Cattolica AUSL della Romagna Ambito Territoriale di Rimini - Italy

## Abstract

**Objectives:**

To obtain outcomes data on anatomical and reverse total shoulder arthroplasty by analysis of clinical scores and standard radiographs.

**Subject selection and enrollment:**

400 consecutive series of patients replaced with anatomical and reverse total shoulder arthroplasty (minimum 3 years follow-up).

**Study Design:**

retrospective monocenter.

**Preoperative assessment:**

Demographics, clinical scores (Constant-Murley) as available, shoulder X-ray (AP, outlet and axillary views) .

**Last follow-up:**

Postoperative radiographhs and clinical scores. Adverse events and complications to be reported as occurred since implantation.

**Statistical analysis:**

Data collected will be summarized and analyzed for statistical significance.

## I. INTRODUCTION

Shoulder arthroplasty is an effective device to treat osteoarthritis and other degenerative conditions of the glenohumeral joint. Modern prosthetic implants allow a total replacement of the shoulder joint to gain a pain free range of motion, and as result this leads to a significant improvement in patient’s quality of life [1–8]. For over 30 years, orthopedic surgeons used anatomical and reverse prosthetic implants that ensured safety and reliability.

Indications for one or the other type of prosthesis depends on the quality of the rotator cuff (RC) tendons, reserving reverse arthroplasty for those with severe insufficiency of the RC [7]. In our Shoulder Unit both type of prostheses have been implanted to treat shoulder osteoarthritis and proximal humeral fractures, but the results previous published refer to small sample size and short-term follow-up.

## II. PURPOSE

Aim of the current study will be to assess clinical and radiological outcomes of anatomical and reverse shoulder arthroplasty wiht a minimum 3 years follow-up (mean 8 years).

## II. PATIENT AND METHODS

### Study Design

The current research project refers to a retrospective observational study on a consecutive series of patients underwent to anatomical and reverse shoulder replacement with the prosthetic devices available at the Unit of Shoulder and Elbow Surgery of D. Cervesi Hospital in Cattolica (Italy) where the same patients were enrolled for preoperative clinical and radiographic exams.

### Study population and enrollment

We foresee to enroll 400 subjects with anatomcial and reverse total shoulder replacement (Zimmer, Tornier, Lima, Biomet) implanted between march 2005 and december 2013, to collect preoperative demographic and clinical data, technical data of intraoperative phase and postoperative clinical and radiographic outcomes at last follow-up.

### Prosthetic design

#### Anatomical

Anatomical total shoulder arthroplasty assessed in this study includes: i) unconstrained monoblock or modular humeral components, ii) unstemmed hydrossyapatite coated “corolla” impacted without cement in the humeral methaphysis (TESS^®^), iii). short stem prostheses with a prevalent methaphysal grip. Head prostheses available in several size, standard or with eccentric offset. Glenoid prostheses including: i) polyethylene components with keel or pegs, fixed in the cancellous bone with cement; the pegged glenoid are also available with a flanged uncemented central peg to promote osseointegration, ii) standard metal-backed glenoid fixed with screw and covered with a polyethylene liner, iii) trabecular tantalium-backed glenoid (TMT^®^) fixed in the bone under pressure.

#### Reverse

Reverse prostheses assessed in this study is a semiconstrained totally modular device. The glenoid component consist of a baseplate (metaglene), provided with a large central peg and secured to the native glenoid by cortical screws (2 or 4), which may be straight or angled, on which is fit the glenosphere (a rounded metal ball approximately two third of sphere) that is attached to the baseplate with a screw. The glenosphere can be completely medialized or slightly lateralized, to prevent scapular neck erosion. The humeral component consists of a proximal cup-shaped portion and a metal stem to be press-fitted or cemented in the medullary canal. A radiolucent polyethylene insert sits in this cup portion and articulates with the glenosphere. As for the anatomical implantants, also for reverse prostheses are available short stem having a predominantely metaphyseal grip.

### Clinical and radiographic evaluation

Preoperative and postoperative clinical outcomes will be evaluated with the Constant-Murley score (CS) [9]. The CS includes a subjective questionnaire for pain, the ability to perform daily living activity (DLA), an objective evaluation of active range of motion (ROM) and strength. Pain will be scored on a 15 points scale (0 severe pain, 15 no pain), while DLA will be scored on a 20 points scale, with lower scores associated with greater impairment on DLA. ROM will be measured using a standard goniometer between the upper arm and the upper part of the thorax. Shoulder strength will be assessed using the Lafayette handheld dynamometer (Lafayette Instruments, Lafayette, Ind, USA), that has a microprocessor with a resolution of 0.4 lb (0.2 kg) in the range 0-50 pounds (0–22.6 kg), 0.03% accuracy with two calibration points: 0.25 and 50 lbs (0.11 and 22.6 kg). Data will be recorded and analyzed using SPSS v.10 software (SPSS Inc, Chicago, IL, USA). We will assign 1 point for each 0.5 kg of strength registered.

Radiographic assessment included standard AP, outlet and axillary views. These radiograms were prescribed when the patient was discharged from Cervesi Hospital to be shown at the first follow-up visit (mean 2 months) and then adviced at 6 months, and every years to assess implants features. Clinical and radiographic data of last follow-up will be compared with those performed annually to detect any pathological features of the implants. In anatomical prostheses the following parameters will be evaluated [10] orientation of the humeral component, translation of the humeral component, offset of the humeral head, size and height of the humeral head, acromio-humeral distance, distribution and fixation of the cement, stress shielding and cortical resorption, radiolucent lines, subsidence and tilt of glenoid and humeral component, glenoid loosening.

Axillary radiograph is the gold standard to assess subluxation of the prosthetic head in sagittal plane that can be classified based on direction and severity as:

Absent: the humeral head is centred in the glenoid cavity
Slight: < 25% translation of the centre of the head component with respect to the glenoid centre
Moderate: 25% to 50% translation of the centre of the prosthetic head with respect to the glenoid centre
Severe: > 50% translation of the centre of the head component with respect to the glenoid centre.

In patient with reverse arthroplasty the following radiographic features will be analyzed: Scapular notching classified according to Nerot et al [11], lucent lines around humerus, baseplate and screws, pillar spur, instability, component disassembly, baseplate mobilizazion or migration.

### Inclusion criteria

Age: 18 years minimum.

Gender: male and female.

Informed Consent - patient or patient’s legal representative has signed a “Patient Informed Consent form”.

TC or MRI to identify concentric osteoarthritis (Samilson grade III and IV) or eccentric osteoarthrits with irreparable rotator cuff tear

### Exclusion criteria

Cognitive limitations that precluded a valid consent to be included in the study

Unwilling to be enrolled

Lack of appropriate patient information

Lost to the last follow-up

### Statistical analysis

Data collected will be summarized descriptively. Summaries will routinely describe categorical data as counts and percentages, and ninety-five percent confidence limits will be generally used to assess differences between different implant configurations. Summaries describing continuous data will be in the form of means, medians, standard deviations, minima, and maxima, and ninety-five percent confidence intervals will be used to contrast differences. Routine summaries of implant survival, return to function, etc. (e.g. time to event) will generally be described via the Kaplan-Meier method and these will generally be accompanied with the corresponding crude rates (expressed as percentages). Routine summaries of complication data will be in the form of frequencies and percentages. Summaries may be further generated for strata within the study population, (e.g. males and females, at different cut-points in the body mass index continuum, etc.). Patient confidentiality will be protected at all times, and patient identifiers will not be included in study summaries.

### Risks and adverse events

As defined by EN ISO 14155-1 and ISO 14155-2.

*Adverse Event*: An “adverse event” is defined as any untoward medical occurrence in a subject. This definition does not imply that there is a relationship between the adverse event and the device under investigation. Adverse event is synonymous with complication or medical event.

*Serious Adverse Event:* A “serious adverse event” is defined as an adverse event that results in death, is lifethreatening, requires inpatient hospitalisation or prolongation of existing hospitalisation, results in persistent or significant disability/incapacity.

*Adverse Device Effect*: An “adverse device effect” is defined as “any untoward and unintended response to a medical device”. This definition includes any event resulting from insufficiencies or inadequacies in the instructions for use or the deployment of the device. This definition includes any event that is a result of a user error.

*Serious Adverse Device Effect*: A “serious adverse device effect” is defined as “an adverse device effect that has resulted in any of the consequences characteristic of a serious adverse event or that might have led to any of these consequences if suitable action had not been taken or intervention had not been made or if circumstances had been less opportune”.

NOTE: The term “severe” refers to the intensity of the event and can be used with any event, without regard to whether or not it meets the criteria for being classified as “serious” or “unanticipated”. For example, a subject can have a severe headache, but it is not a serious event.

### Reporting of Adverse Events and Adverse Device Effects

All adverse events which occur during the study will be reported and will identify the following:

- Description of symptoms
- Date of onset
- Severity of symptoms: mild, moderate, severe
- Relation to device: not related, uncertain, probably, definitely
- Treatment
- Outcome of treatment: resolved, tolerated, pending, study withdrawal, device removed/re-operation, death
- Additional comments

Ethical considerations

### Patient Information and Informed Consent

The investigator must explain to each patient the nature of this retrospective study, including any risks and benefits, its purpose and procedures and expected duration of involvement in the study. Patients have full rights to withdraw from the study at any time, irrespective of their initial consent.

### Subject Confidentiality

Confidentiality of patient data will be maintained at all times. Patient anonymity will be guaranteed and all documentation relating to a subject will be kept in a secure location.

### Declaration of Helsinki

This study will be conducted in accordance with the relevant articles of the Declaration of Helsinki as adopted by the 18th World Medical Assembly in 1964 and as revised in Tokyo (1975), Venice (1983), Hong Kong (1989), South Africa (1996) and Edinburgh (2000).

## References

[1] Merolla G, Porcellini G (2014). Reverse Shoulder Arthroplasty in Patients Aged Sixty Years Old or Younger: Are we Really Doing the Best?. Transl Med UniSa.

[2] Merolla G, Nastrucci G, Porcellini G (2013). Shoulder arthroplasty in osteoarthritis: current concepts in biomechanics and surgical technique. Transl Med UniSa.

[3] Merolla G, Paladini P, Campi F, Porcellini G (2008). Efficacy of anatomical prostheses in primary glenohumeral osteoarthritis. Chir Organi Mov.

[4] Merolla G, Campi F, Paladini P, Lollino N, Fauci F, Porcellini G (2009). Correlation between radiographic risk for glenoid component loosening and clinical scores in shoulder arthroplasty. Chir Organi Mov.

[5] Mizuno N, Denard PJ, Raiss P, Walch G (2013). Reverse total shoulder arthroplasty for primary glenohumeral osteoarthritis in patients with a biconcave glenoid. J Bone Joint Surg Am.

[6] Flurin PH, Marczuk Y, Janout M, Wright TW, Zuckerman J, Roche CP (2013). Comparison of outcomes using anatomic and reverse total shoulder arthroplasty. Bull Hosp Jt Dis.

[7] Nolan BM, Ankerson E, Wiater JM (2011). Reverse total shoulder arthroplasty improves function in cuff tear arthropathy. Clin Orthop Relat Res.

[8] Muh SJ, Streit JJ, Wanner JP (2013). Early follow-up of reverse total shoulder arthroplasty in patients sixty years of age or younger. J Bone Joint Surg Am.

[9] Constant CR, Murley AH (1987). A clinical method of functional assessment of the shoulder. Clin Orthop Relat Res.

[10] Merolla G, Di Pietto F, Romano S, Paladini P, Campi F, Porcellini G (2008). Radiographic analysis of anatomical shoulder arthroplasty. Eur J Radiol.

[11] Valenti P, Boutens D, Nerot C, Walch G, Boileau P, Molé D (2001). Delta 3 reversed prosthesis for osteoarthritis with massive rotator cuff tear: long term results (>5 years). Shoulder prosthesis.

